# The importance of an interaction network for proper DNA polymerase ζ heterotetramer activity

**DOI:** 10.1007/s00294-017-0789-1

**Published:** 2017-11-30

**Authors:** Ewa Szwajczak, Iwona J. Fijalkowska, Catherine Suski

**Affiliations:** Institute of Biochemistry and Biophysics, Polish Academy of Sciences, Pawinskiego 5a, 02-106 Warszawa, Poland

**Keywords:** DNA polymerase zeta (Pol ζ), CysB motif, Translesion synthesis, Mutagenesis, *Saccharomyces cerevisiae*

## Abstract

Precisely controlled mechanisms have been evolved to rescue impeded DNA replication resulting from encountered obstacles and involve a set of low-fidelity translesion synthesis (TLS) DNA polymerases. Studies in recent years have brought new insights into those TLS polymerases, especially concerning the structure and subunit composition of DNA polymerase zeta (Pol ζ). Pol ζ is predominantly involved in induced mutagenesis as well as the bypass of noncanonical DNA structures, and it is proficient in extending from terminal mismatched nucleotides incorporated by major replicative DNA polymerases. Two active forms of Pol ζ, heterodimeric (Pol ζ_2_) and heterotetrameric (Pol ζ_4_) ones, have been identified and studied. Here, in the light of recent publications regarding induced and spontaneous mutagenesis and diverse interactions within Pol ζ holoenzyme, combined with Pol ζ binding to the TLS polymerase Rev1p, we discuss the subunit composition of Pol ζ in various cellular physiological conditions. Available data show that it is the heterotetrameric form of Pol ζ that is involved both during spontaneous and induced mutagenesis, and underline the importance of interactions within Pol ζ when an increased Pol ζ recruitment occurs. Understanding Pol ζ function in the bypass of DNA obstacles would give a significant insight into cellular tolerance of DNA damage, genetic instability and the onset of cancer progression.

Living cells are continuously exposed to stressful conditions, with their genomes constantly treated by environmental and endogenous DNA damaging agents (Halas et al. 2009; Ruiz-Roig et al. 2010; Yosef et al. 2016; Chalissery et al. 2017; Roy 2017). Lack of efficient tolerance pathways for such damaged DNA may lead to replication stress and in consequence to genomic instability and cancer (Sharma et al. 2013; Zeman and Cimprich 2014; Gaillard et al. 2015; Adamczyk et al. 2016; Jeggo et al. 2016). The translesion synthesis (TLS) DNA polymerases zeta (Pol ζ) enables bypass of damaged templates as well as replication through noncanonical DNA structures, which cannot be bypassed by major DNA replicases, although often at the cost of increased mutagenesis (for a review, see Makarova and Burgers 2015; Vaisman and Woodgate 2017). The replicative bypass by TLS polymerases is usually performed in a two-step process assisted by the monoubiquitinated proliferating cell nuclear antigen (PCNA) sliding clamp (reviewed in Vaisman and Woodgate 2017; Zhao and Washington 2017). In *Saccharomyces cerevisiae*, during the Pol ζ-dependent damage bypass, the first step is often performed by the inserter Y-family TLS DNA polymerase, Rev1p (Washington et al. 2004; Kim et al. 2011; Wiltrout and Walker 2011; Northam et al. 2014). Rev1p possesses deoxycytidyl transferase activity and is, therefore, responsible for the high frequency of “C” incorporation opposite encountered lesions (Nelson et al. 1996a). The second step is accomplished by Pol ζ, which has the ability to proficiently extend aberrant primer termini, thereby contributing to the fixation of mutations (Johnson et al. 2000; Haracska et al. 2003). Furthermore, during DNA replication, the major DNA polymerases may also incorporate mismatched nucleotides, what may lead to their dissociation from the replication fork and subsequent exchange with Pol ζ error-prone polymerase, which extends the mispaired primer termini (for review see Shcherbakova and Fijalkowska 2006; Pavlov and Shcherbakova 2010; Sale 2012). Pol ζ mutagenicity can also result from an increased frequency of incorrect base insertions, due to its low nucleotide selectivity (Arana and Kunkel 2010) and a lack of 3ʹ→5ʹ exonuclease proofreading activity (Nelson et al. 1996b). Therefore, the error-prone Pol ζ plays a predominant role in both the spontaneous mutagenesis during normal cell growth, when cells are not treated with any external damaging agents (Quah et al. 1980) as well as damage-induced mutagenesis (for a review, see Lawrence 2002; Vaisman and Woodgate 2017; Zhao and Washington 2017). Indeed, in wild-type *S. cerevisiae* cells, the deletion of the Pol ζ catalytic subunit has an antimutator effect and eliminates 50–70% of spontaneous and over 90% of damage-induced mutagenesis (Cassier et al. 1980; Quah et al. 1980; Roche et al. 1994; Lawrence 2002; Sabbioneda et al. 2005; Northam et al. 2006, 2010; Halas et al. 2009; Kraszewska et al. 2012; Grabowska et al. 2014; Garbacz et al. 2015).

Studies in vitro have shown that the minimally functional Pol ζ complex (Pol ζ_2_) is composed of two subunits, where the catalytic Rev3p subunit physically interacts via its N-terminal region with the auxiliary Rev7p subunit (Nelson et al. 1996b). Recent studies showed that Pol31p-Pol32p, two subunits of the major replicative lagging-strand DNA polymerase Pol δ, were purified along with Rev3p-Rev7p to form a fully functional four-subunit complex, Pol ζ_4_ (Rev3p, Rev7p, Pol31p and Pol32p) (Fig. 1a) (Baranovskiy et al. 2012; Johnson et al. 2012).

**Fig. 1:**
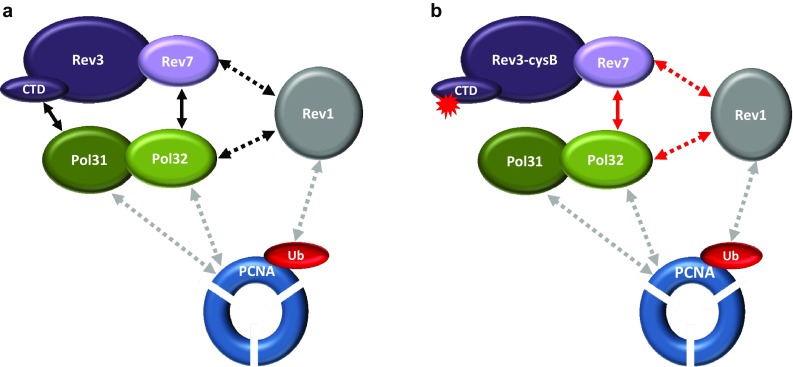
The interaction network between Pol ζ heterotetramer, Rev1p and PCNA. **a** Interactions within Pol ζ_4_ are indicated by continuous line, dotted lines indicates interactions of particular Pol ζ_4_ subunits with Rev1p (dark grey) and PCNA (light grey). **b** Alteration of interactions in Rev3-cysBp Pol ζ_4_ holoenzyme. Pol32p-Rev7p interaction and further interactions of Pol32p and Rev7p with Rev1p (all marked in red) create the opportunity for partial reconstitution of Pol ζ_4_ holoenzyme when the Rev3p-Pol31p binding is abolished. See text for details

The C-terminal domain (CTD) of the catalytic subunit of Pol ζ, Rev3p, shows strong sequence homology in two conserved cysteine-rich metal-binding motifs, CysA and CysB, with the C-terminal domain of the Pol3p catalytic subunit of Polδ (Netz et al. 2011) and is responsible for the interaction with Pol31p (Fig. 1a), which in turn serves as a matchmaker with the Pol32p subunit (Gerik et al. 1998). However, while the intact CysA and CysB motifs are both required for proper DNA replication by Pol δ (Netz et al. 2011), only mutations in the CysB motif of Rev3p abolish Rev3p-Pol31p binding (Baranovskiy et al. 2012; Johnson et al. 2012; Makarova et al. 2012). The CysB motif of Rev3p coordinates an iron-sulfur [4Fe-4S] cluster, required for the specific interaction with Pol31p. Substitution of even two of the four conserved cysteines in the CysB motif disrupts the iron-sulfur [4Fe-4S] cluster, preventing specific Rev3p-Pol31p binding and thus the proper Pol ζ_4_ heterotetramer formation (Fig. 1b) (Makarova et al. 2012). Notably, the Rev3p–Rev7p interaction is not affected in such mutant strain (Makarova et al. 2012).

In vivo experiments have shown a severe decrease in UV-induced mutagenesis in strains carrying the mutated CysB motif, strongly suggesting that Pol ζ_4_ is required for damage-induced mutagenesis (Baranovskiy et al. 2012; Johnson et al. 2012; Makarova et al. 2012; Siebler et al. 2014). Recently, employing the *rev3-cysB* allele (*rev3-CC1449,1473SS* (Makarova et al. 2012)), we analyzed which Pol ζ form, Pol ζ_4_ or Pol ζ_2_, is engaged in spontaneous mutagenesis in budding yeast using the *CAN1* reporter gene, which enables the simultaneous detection of a wide spectrum of mutational events (Chen and Kolodner 1999). In addition to the wild-type strain, we studied strains possessing mutations affecting the catalytic subunits of the major replicative polymerases (*pol3-Y708A* and *pol2-1*) (Morrison et al. 1990; Pavlov et al. 2001), which lead to defective DNA replication and in turn to more frequent Pol ζ recruitment and an increase of spontaneous mutation rates (Northam et al. 2006), a phenomenon called DRIM (defective-replisome-induced mutagenesis) (Northam et al. 2010). Previously, we and others also showed that defects in the non-catalytic components of the replisome may lead to a Pol ζ-dependent mutator phenotype (Northam et al. 2006; Aksenova et al. 2010; Kraszewska et al. 2012; Becker et al. 2014; Grabowska et al. 2014; Garbacz et al. 2015). In Szwajczak et al. (2017), we studied a *psf1-100* strain possessing the mutated Psf1p subunit of the GINS complex (Grabowska et al. 2014). In this mutant strain, the interaction between the major DNA helicase and the leading strand DNA polymerase Pol ε is impaired (Grabowska et al. 2014), which may affect leading strand replication. A defective replisome may stall at noncanonical DNA structures, which can be proficiently bypassed by Pol ζ supported by Rev1p (Northam et al. 2014). Thus, the DRIM phenotype observed in the studied strains results from the increased participation of Pol ζ in the synthesis of undamaged DNA (Northam et al. 2010, 2014). Our studies indicated that in defective replisome strains (*pol3-Y708A, pol2-1, psf1-100*), a majority of arising mutations can be attributed to the heterotetrameric form of Pol ζ (Szwajczak et al. 2017).

The fact that Pol31p and Pol32p are accessory subunits of two different polymerases, the major replicase Pol δ and the error-prone Pol ζ (Baranovskiy et al. 2012; Johnson et al. 2012; Makarova et al. 2012), seems crucial for Pol ζ recruitment to the replication fork. A switching mechanism was proposed between Pol ζ and Pol δ through an exchange of the catalytic subunits on Pol31p-Pol32p bound to PCNA (Baranovskiy et al. 2012). In this scenario, as a result of replication impediments, Pol3p dissociates, and the Rev3p-Rev7p heterodimer is recruited to the Pol31p-Pol32p remaining at the primer terminus (Baranovskiy et al. 2012). This hypothesis is perfectly consistent with lagging strand synthesis, where Pol δ provides replicative synthesis. In *pol2-1* or *psf1-100* strains it is reasonable to propose that the leading strand synthesis performed by Pol ε is impaired. However, it remains unknown whether Pol ζ is recruited in such mutant strains as a result of exchange between Pol ε and Pol ζ or whether the Pol ε-Pol δ switch may occur prior to Pol ζ recruitment. Additionally, in the *pol2-1* mutant strain, a structural defect of Pol2p may trigger a more global replication impairment and consequently increase Pol δ involvement in the leading strand synthesis (reviewed in Pavlov and Shcherbakova 2010; Stillman 2015). Indeed, we have found a synergistic relationship in spontaneous mutagenesis rates between pol3-5DV, in which the intrinsic Pol δ proofreading activity is inactivated (Jin et al. 2001), and *pol2-1*, suggesting the increased participation of Pol δ in the *pol2-1* mutant strain (unpublished data). Therefore, in the *pol2-1* strain, Pol ζ recruitment could result from a switch between Pol δ and Pol ζ.

In contrast, in the wild-type strain, the *rev3-cysB* mutation decreases the level of spontaneous mutagenesis, but not to the same extent as the *REV3* deletion (*rev3Δ*) (Szwajczak et al. 2017). This moderate though statistically significant difference was observed in several different genetic backgrounds (Szwajczak et al. 2017), thus may be considered as meaningful (Behringer and Hall 2016). The intermediate antimutator effect might suggest that a part of the mutagenesis observed in the *rev3-cysB* mutant strain is a result of Pol ζ_2_ involvement, for which activity has been shown in vitro, although this activity was much weaker than that of Pol ζ_4_ (Makarova et al. 2012). Pol31p and Pol32p subunits are required for the direct functional interaction between Pol ζ_4_ and PCNA (Fig. 1a), which enhances Pol ζ-mediated TLS (Makarova et al. 2012). However, in the absence of Pol31p and Pol32p subunits, the Pol ζ_2_ interaction with PCNA may still be mediated *via* Rev1p, which binds both to Rev7p and monoubiquitinated PCNA (Fig. 1a) (Acharya et al. 2005; Wood et al. 2007). Another possibility is that even in the presence of the *rev3-cysB* mutation, other protein–protein interactions within the Pol ζ holoenzyme as well as interactions with Rev1p may at least partially restore functional Pol ζ_4_ (Fig. 1b). Indeed, the Rev7p-Pol32p interaction may stabilize the Pol ζ_4_ heterotetramer in the absence of Rev3p-Pol31p binding. Furthermore, Rev1p, interacting with both Rev7p and Pol32p (Acharya et al. 2005, 2009), may serve as an additional stabilizer of such reconstituted Pol ζ_4_ complex. In either case, the structural role of Rev1p, crucial for Pol ζ-dependent mutagenesis (Nelson et al. 2000), may be of even greater importance in strains with impaired interactions within the Pol ζ_4_ holoenzyme.

The potential reconstitution of the Pol ζ_4_ tetramer via the Pol32p-Rev7p interaction in the *rev3-cysB* mutant strain is not possible in Pol32p-deficient cells. Pol32p plays an important role as Pol ζ subunit, as abolition of Pol ζ-dependent damage-induced mutagenesis is observed in strain with *POL32* deletion (*pol32Δ*) (Gerik et al. 1998). Intriguingly, in the *pol32Δ* strain, no decrease in the spontaneous mutagenesis rate is observed (Huang et al. 2002) (Table 1). However, *REV3* deletion does not exert such an antimutator effect in *pol32Δ* as in Pol32p-proficient strain (Table 1), which may signify that the majority of Pol ζ-dependent mutagenesis is already abolished in *pol32Δ* strain. This result supports the in vitro finding that Pol32p is required for stable Rev3p-Pol31p complex formation (Makarova et al. 2012). Interestingly, we observed that *pol32Δ* evens the level of spontaneous mutagenesis in the *rev3-CysB* and *rev3Δ* mutant strains (56 × 10^−8^ in *rev3Δ pol32Δ* and 55 × 10^−8^ in *rev3-cysB pol32Δ*) (Table 1), suggesting that noticeable Pol ζ-dependent mutagenesis in the *rev3-cysB* strain is due to the alternative reconstitution of Pol ζ_4_, rather than Pol ζ_2_ activity. Unfortunately, it is difficult to compare spontaneous mutagenesis levels between *rev3Δ* and *pol32Δ* strains, as Pol32p not only plays a single role as Pol ζ subunit but additionally participates in reactions proceeded by Pol δ. Indeed, the level of spontaneous mutagenesis in *pol32Δ* is higher than that in the *rev3Δ* strain (Table 1); interestingly, in relation to *pol32Δ* cold-sensitivity (Huang et al. 2000), this level is even more increased at lower temperatures (twofold increase of mutagenesis rate in *pol32Δ* at 23 °C, compared to 30 °C, data not shown). Thus, *pol32Δ* may not exhibit an antimutator phenotype, in contrast with *rev3Δ*, as the diminution of Pol ζ-dependent mutagenesis is compensated by a moderate mutator phenotype related to the involvement of Pol32p in Pol δ reactions. The role of Pol32p in the proper formation of Pol ζ may also be supported by previous studies (Johansson et al. 2004), where the authors showed that the elimination of the physical interaction between Pol31p and Pol32p decreases the level of UV-induced mutagenesis. This result emphasizes not only the role of Pol32p in PCNA binding, but also in the proper formation of Pol ζ heterotetramer.

**Table 1:** Spontaneous mutation rates for wild-type, *rev3Δ* and *rev3-cysB* in Pol32p-proficient and Pol32p-deficient backgrounds

Relevant genotype	Can^R^ (× 10^−8^)	
Pol32p-proficient strains		
*REV3*	63	(59–68)
*rev3Δ*	30	(27–34)
*rev3-cysB*	47	(44–49)
Pol32p-deficient strains		
*REV3 pol32Δ*	68	(60–72)
*rev3Δ pol32Δ*	56	(51–63)
*rev3-cysB pol32Δ*	55	(48–66)

The yeast strains used in this study were constructed in the SC765 background (Grabowska et al. 2014), derivative of ΔI(-2)I-7B-YUNI300 (Pavlov et al. 2002). The rates of spontaneous mutagenesis were determined using the *CAN1* reporter gene, enabling the simultaneous detection of a wide spectrum of mutational events (Chen and Kolodner 1999). The experiments were performed as described in Szwajczak et al. (2017). The 95% confidence intervals are shown in parentheses; *p* values between corresponding strains were calculated using a non-parametric Mann–Whitney *U* test. Statistically significant differences (*p* values < 0.05) were observed between the following pairs of strains: *REV3 vs. rev3Δ, REV3 vs. rev3-cysB, rev3Δ vs. rev3-cysB, rev3Δ vs. REV3 pol32Δ, rev3Δ vs. rev3Δ pol32Δ, rev3Δ vs. rev3-cysB pol32Δ, rev3-cysB vs. REV3 pol32Δ, rev3-cysB vs. rev3Δ pol32Δ, rev3-cysB vs. rev3-cysB pol32Δ* and *REV3 pol32Δ vs. rev3Δ pol32Δ*

In the wild-type strain, in which the frequency of Pol ζ recruitment is lower than that under DRIM conditions, impaired Rev3p-Pol31p binding may be partially compensated by other interactions within the Pol ζ_4_ holoenzyme. Such reconstitution may be less efficient in defective replisome strains, in which mutations in the CysB motif eliminate a majority of spontaneous mutagenesis (Szwajczak et al. 2017). Similarly, some fraction of mutagenesis was still observed in strains with a mutated CysB motif after minimal UV exposition, while at higher UV doses, the difference in mutagenesis rates between strain with CysB mutations and *rev3Δ* was diminished (Siebler et al. 2014). Additionally, we observed that the survival rate in the *rev3-cysB* mutant strain is higher than that in strain with *rev3Δ* at a lower UV dose (48% compared to 24% at 5 J/m^2^, unpublished data), whereas at higher doses, mutations in the CysB motif and the deletion of *REV3* comparably affect the survival rate (Baranovskiy et al. 2012; Johnson et al. 2012; Makarova et al. 2012; Siebler et al. 2014, our unpublished data). Siebler et al. (2014) suggested that this effect indicates that the mechanism of TLS may be somewhat dependent on the level of DNA damage. This hypothesis might also be applicable to spontaneous mutagenesis conditions. When the frequency of replication impediments is substantially increased (DRIM), this recruitment of heterotetrameric Pol ζ to DNA may be under a more precise control. Moreover, interactions with some new players or post-translational modifications may additionally stabilize the Pol ζ structure.

Mutual interactions within the Pol ζ_4_ holoenzyme and between Pol ζ_4_ subunits and two scaffold proteins, Rev1p and PCNA, create an intricate connection network (Fig. 1a). This precise pattern of interactions may thus stabilize Pol ζ holoenzyme and influence its recruitment onto DNA. Based on available data, we suggest that the recruitment of a stable Pol ζ_4_ may be especially required when the involvement of Pol ζ is increased due to destabilized replisome or higher level of DNA damage. However, Pol ζ recruitment and control still needs to be thoroughly investigated, especially in conditions of increased Pol ζ involvement in DNA replication, which may lead to genetic instability and cancer (Knobel and Marti 2011; Lange et al. 2011, 2013; Skoneczna et al. 2015; van Loon et al. 2015; Korzhnev and Hadden 2016; Vaisman and Woodgate 2017; Zhao and Washington 2017). Further uncovering of Pol ζ role in various cellular processes could shed new light on cancer development and evolution processes.
